# Validation of the Partners at Care Transitions Measure (PACT-M): assessing the quality and safety of care transitions for older people in the UK

**DOI:** 10.1186/s12913-020-05369-1

**Published:** 2020-07-01

**Authors:** Eirini Oikonomou, Bethan Page, Rebecca Lawton, Jenni Murray, Helen Higham, Charles Vincent

**Affiliations:** 1 University of Oxford, Oxford, UK; 2 School of Psychology, University of Leeds, Leeds, UK; 3 Bradford Institute For Health Research, Bradford, UK

**Keywords:** Transition, Care, Health, Measure, Tool, Validation, Reliability, patient safety, Quality, Older patients

## Abstract

**Background:**

The Partners at Care Transitions Measure (PACT-M) is a patient-reported questionnaire for evaluation of the quality and safety of care transitions from hospital to home, as experienced by older adults. PACT-M has two components; PACT-M 1 to capture the immediate post discharge period and PACT-M 2 to assess the experience of managing care at home. In this study, we aim to examine the psychometric properties, factor structure, validity and reliability of the PACT-M.

**Methods:**

We administered the PACT-M over the phone and by mail, within one week post discharge with 138 participants and one month after discharge with 110 participants. We performed principal components analysis and factors were assessed for internal consistency, reliability and construct validity.

**Results:**

Reliability was assessed by calculating Cronbach’s alpha for the 9-item PACT-M 1 and 8-item PACT-M 2 and exploratory factor analysis was performed to evaluate dimensionality of the scales. Principal components analysis was chosen using pair-wise deletion. Both PACT-M 1 and PACT-M 2 showed high internal consistency and good internal reliability values and conveyed unidimensional scale characteristics with high reliability scores; above 0.8.

**Conclusions:**

The PACT-M has shown evidence to suggest that it is a reliable measure to capture patients’ perception of the quality of discharge arrangements and also on patients’ ability to manage their care at home one month post discharge. PACT-M 1 is a marker of patient experience of transition and PACT-M 2 of coping at home.

## Background

Care transition is a sequence of activities before and after discharge, designed to ensure coordination between providers and continuity of care between services, as patients move across health care services [1]. Most transitions require considerable and accurate cooperation between various health services providers and clinical teams; the hospital, the primary care team, social services, home care clinicians, community health professionals, the patient and their relatives or carers [2]. For older, more vulnerable adults with multiple chronic conditions the transition of care from hospital to home is a complicated process often associated with inadequate communication between providers, lack of patient involvement, delays in service provision and limited follow-up [3]. Risk factors such as; patients’ individual needs, level of frailty, support network, accessing health services and social barriers [3–5] contribute to the decrease of overall health [1, 6].

Findings from multiple studies indicate that older adults experience more frequent interactions with health care professionals including emergency department visits and hospital admissions that increase the risk of safety incidents occurring during the transfer across settings and between providers [1, 3, 7]. Older adults transferred from hospital to home with more complex health needs are in greater risk to experience safety incidents such as unplanned readmissions within the first thirty days post discharge when compared to the general population [8–10]. Safety incidents and adverse events are often related to; insufficient clinical information, medication errors and poor communication between the hospital and primary and community care services. Adverse events during transitions have been observed to occur to one in five patients [11–13] and one in six patients over 75 years old are readmitted to hospital within the first month post discharge [14].

Improving the quality of transition relies on developing reliable measurement tools that are scientifically rigorous, evidence based, enable monitoring of safety markers, require easily collected data using available resources, help health services providers to see safety through the eyes of patients and outcomes can be used as a common language of service quality across the organisation [15–17]. For instance, the Medical Home Care Coordination Survey (MHCCS) [18] has been developed to assess key components of care continuity (e.g. relationships with providers in hospital, communication of information to patients, post hospital care etc.) from patient’s perspective and has been used as a quality indicator of discharge care [19]. A systematic review by Doyle et al. [20] showed that patient experience self-report measures are a reliable marker of the quality of health services and have been positively associated with patient safety and clinical efficiency for a wide array of health problems and clinical settings, including discharge care. Patient experience surveys have been shown to objectively evaluate clinical outcomes such as continuity of care, compliance with clinical practice guidelines, coordination between professionals and adverse events [20]. Patient reports of the quality of transition obviously reflect their own experience but could be a reasonable reflection of the quality and wider effectiveness of the transition process.

Patient experience is important in understanding the quality of discharge care as it reveals strengths and weaknesses in the safety of services. Patients and caregivers may not know who is coordinating their care needs [21, 22], which health service to contact for advice [23], often struggle to manage their condition and symptoms after discharge [24] and feel frustrated by not receiving clear medication directions [23]. Nevertheless, qualitative accounts of patients followed throughout care transitions showed that high level of comprehension of the information around their health at discharge does not ensure ongoing capability to cope with health and manage symptoms at home weeks after discharge [25].

The Care Transition Measure (CTM) [26] is the most common tool to evaluate transition experience from hospital to home. Although the CTM is a validated instrument designed to assess various components of transitional quality (for example, information transfer, patient empowerment, patient and caregiver preparation, etc.), it only focuses on the immediate post discharge period and does not include safety related components. Other tools measuring quality of care transitions using patients’ experiences have covered either a single area of transition, such as discharge, readmission or referral, or related concepts like care co-ordination, home care, continuity of care and patient satisfaction [18, 22, 26–37]. These measures have been designed to address transition challenges of a target population and healthcare system different to the UK (for example, US citizens) and have not included the concept of patient safety across the continuum of transitional care; from the immediate to the longer-term post discharge period.

**Fig. 1:**
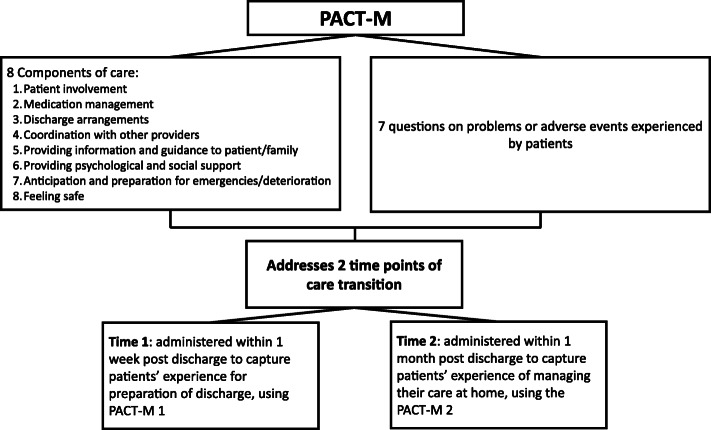
The PACT-M

The PACT-M [38] (Fig. 1) is the only measure developed within the UK health system, that assesses patient experience, quality and safety of care transition. The PACT-M addresses both the immediate post-discharge period and the longer-term experience of managing health and care at home. The development of the PACT-M was an iterative process which has been described elsewhere [38]. The main development stages involved: constructing a theoretical framework, identifying key areas of transitions, designing an item pool to address these areas, evaluating the items through Delphi groups and finally, pilot testing of the measure [38].

### Aims of the present study

1. To perform a preliminary assessment of the transition experience for older patients as they move from hospital to home.
2. To explore the factor structure of the PACT-M 1 and PACT-M 2.
3. To assess the scale’s psychometric properties, particularly inter-item correlations and establish the validity and reliability of the PACT-M. Specifically, the present study seeks to to test the internal reliability of the measure using Cronbach’s alpha criterion.

## Methods

### Study participants

Participants were recruited whilst they were in hospital, over a period of six months. We based the study in a large teaching NHS Foundation Trust in a sample of wards. Our sampling plan included identifying a series of wards caring for older people. These were; cardiothoracic, vascular, surgical, women centre, complex medical care, acute, trauma, stroke, infectious diseases and tropical medicine wards. Eligible patients were identified by senior nurses and were approached for inclusion in the study if the initial interview did not interfere with routine care. Then, the researcher in the field approached potential participants to discuss the study. Inclusion criteria were: English speaking population aged 65 years or older, due to be discharged home, able to provide informed consent, having spent at least one overnight stay in hospital.

This study received ethical approval from the NHS London - Chelsea Research Ethics Committee (ref: 18/LO/0568).

**Fig. 2:**
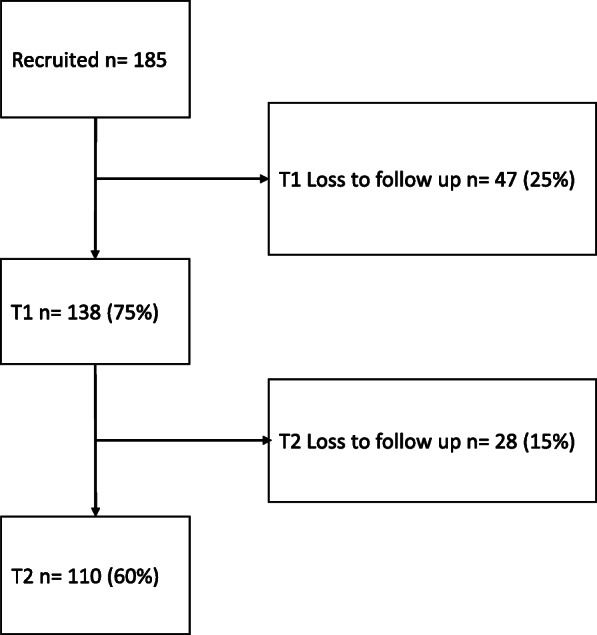
Recruitment flowchart

Participants were given the information sheet and consent form and once they agreed to participate, the study researcher (EO) collected demographic information about their age, gender, ethnicity, estimated discharge date and who they live with (Table 1). The researcher informed participants that they would be contacted at two time points post-discharge and offered the option to be contacted by phone or by post. Only participants that were followed-up at time 1 were also followed-up at time 2 (Fig. 2).

### The measure

The PACT-M includes a survey designed to address eight components about the patient’s experience of transition of care from hospital to home (see Fig. 1 for the full list of components) [38]. In addition, PACT-M involves a list of seven questions in order to capture potential problems with care and adverse events that are commonly experienced by recently discharged patients [38] (see Supplementary file 1, Table 1 and Supplementary file 2, Table 2).

### Measure administration

The majority of participants were contacted by phone. The researchers (EO, BP) performed short phone calls during which, participants were asked to complete the PACT-M. Participants were requested to rate the survey items on an agreement scale (1 to 5 where 1 is strongly disagree and 5 is strongly agree) with the option to choose that the item is ‘not applicable’ and then, they were asked to respond with yes or no answers about potential problems or adverse events with managing their health and care at home. Researchers attempted to call participants up to 3 times on 3 different days and if participants did not answer their phone, they were withdrawn from the study. Five participants, those with more complex health issues or with hearing difficulties indicated they wished to be contacted by post and the questionnaire was sent to the preferred address with a pre-paid, return envelope.

The role of the interviewer is crucial in ensuring low rates of missing values from non-responses; interviewers who are well trained and regularly supervised report low non-responses [39] when administering surveys. For the present study, both interviewers (EO, BP) were trained before administering PACT-M, had regular communication and received supervision from the principal investigator (CV), to discuss any issues or concerns. This enabled the interviews to run smoothly. Once participants had agreed to take part, they almost invariably were willing to complete the interview.

### Statistical analyses

Analyses were conducted using IBM SPSS Statistics 25 Software Package [40]. Descriptive statistics and Pearson’s correlations were calculated for all items. We benchmarked correlations against similar work [41] and we defined a strong correlation as 0.70, moderate to substantial as 0.30–0.70 and weak as 0.30 or below.

There were very few (under 0.01%) missing values at time 1 and no missing values at time 2, as the surveys were completed by the project researchers while on the phone with participants or after receiving participants’ answers by mail.

For the purposes of this analysis, we followed the sample size strategy outlined Devellis’ work [42] which states that there is one rule of thumb for calculating acceptable sample size for factor analysis; a ratio of at least 10:1 between the number of participants and the number of items in a survey, with an ideal ratio being 15:1 or 20:1 [41, 43–46]. In our study, the minimum sample size for factor analysis was satisfied, with a ratio of 14 participants per item for PACT-M 1 and 13 participants per item for PACT-M 2.

Reliability was assessed by calculating Cronbach’s alpha for PACT-M 1 and PACT-M 2. Exploratory factor analysis was conducted to evaluate dimensionality of the scales.

Principal components analysis was chosen, to examine the factor structure of the measure on correlation matrices, using pair-wise deletion. Principal components analysis was chosen due to the exploratory nature of the PACT-M. Kaiser’s criterion was used for choosing the number of factors to retain, as it has been shown to be relatively accurate for samples sizes above 100. Subscales resulting from this analysis were assessed for reliability using Cronbach’s alpha.

## Results

### Participant characteristics

In total, 70% of people approached agreed to take part in the study, of whom, 138 completed PACT-M 1 (response rate 75%) and 110 responded to PACT-M 2 (response rate 60%).

The mean age of the sample (*n* = 138) was 79 years (range: 65–95). The majority of participants were male (59%), of Caucasian, British origin (98%) and living with their spouse or partner (69%). Most participants (73%) were recruited from the cardiothoracic/vascular and surgical emergency wards and almost half of participants had been hospitalised for 5 days or less at the point of consent. Further demographic characteristics are presented in Table 1.

**Table 1:** Participants’ characteristics

	*N* = 138
	%
Gender	
Female	41
Male	59
Age	
65–74	31
> = 75	69
Ethnic group	
White English, Welsh, Scottish, Northern Irish or British	98
Any other white background	1
White and Asian	1
Ward Type	
Cardio/Cardiothoracic/Vascular	37
Vascular	5
Women Centre	12
Surgical	6
Trauma	4
Surgical Emergency	31
Complex Medical Care Unit	3
Stroke	1
Infectious diseases and tropical medicine	3
Living situation	
Live Alone	28
Live with spouse/partner	69
Live with other family member	4

The majority of participants (80%) were contacted within the first week after discharge. Participants that reported feeling unwell, requested to be contacted at a later point or participants that have been readmitted to the hospital but were keen to take part in the study were contacted later than one week from their first discharge. Ninety percent of respondents were contacted within 10 days after their return home, and one participant was contacted 48 days post discharge.

### Patients’ experiences on the preparation for transition PACT-M 1

Table 2 depicts the 9 items of the PACT-M 1 frequencies. The percentage of participants that used the response options ‘strongly disagree’ varied from 0.7 to 13% per item and varied from 20 to 48% per item for the response option ‘strongly agree’. The percentage of participants that used the response options ‘not applicable’ varied from 0 to 33% per item; items 2 and 5, referring to patients being aware of further help and support when at home (such as; home care or community care arrangements, help with cooking, showering, grocery shopping etc.) received the highest ‘not applicable’ scores (23 and 33% respectively). There were almost no missing values (0.01%).

Item mean scores ranged between 3.59 and 4.37 with a mean of 4.1 and mean SD = 0.9. Seven out of nine items were answered by 90% or more participants (i.e. all participants gave a response other than not applicable). The frequency of responses showed the vast majority of participants (90%) felt prepared to be at home. Nine out of ten patients felt they could ask staff questions about their care at home and felt confident they understood how to manage their medications. More than 80% of participants felt ‘they knew what to do if their health deteriorated’, and agreed they could discuss their worries with hospital staff, and any concerns they might have, had been addressed before leaving the hospital. However, 20% of participants did not feel that staff helped them to prepare for non-medical needs at home (e.g. cooking, grocery shopping, showering) and more than one in ten participants did not know about home/community care arrangements made to support them at home and did not know how to get help from out-of-hospital services (e.g. GP, community care) if they needed to.

**Table 2:** Means, standard deviations and frequencies of PACT-M 1 items

**PACT-M 1** ***n*** **= 138**	**Strongly Disagree** **(%)**	**Disagree** **(%)**	**Neither Agree nor Disagree** **(%)**	**Agree** **(%)**	**Strongly Agree** **(%)**	**Not Applicable(%)**	**Missing** **(%)**
1. I felt I could ask staff questions about what will happen after going home.	1	3	6	57	33	1	1
2. Before leaving the hospital, I was confident I understood how to manage my medication.	0	5	2	41	48	4	1
3. While I was in hospital, staff helped me to prepare for things that I might find difficult when I go back home (such as walking, cooking, showering, grocery shopping or being in pain).	4	16	6	30	20	32	1
4. Before leaving the hospital, I understood how to get help (or support) from my community services (e.g. doctors, nurses, home care staff).	2	10	13	39	25	15	0
5. Before leaving the hospital, I knew what arrangements had been made to support me at home (for example home care, community care visits).	0	13	8	19	27	46	0
6. While I was in hospital, there was someone who I could talk to if I was worried.	0	4	2	50	40	5	0
7. Before leaving the hospital, I felt confident about what to do if my health became worse at home.	0	9	2	48	41	0	0
8. I feel that my concerns around my health had been addressed before I went home.	0	7	7	50	34	2	0
9. I feel prepared to be at home.	2	5	4	57	33	0	1

### Patients’ experiences with managing their health and care at home: PACT-M 2

Table 3 shows the 8 PACT-M 2 item frequencies. The percentage of participants that used the response option ‘strongly agree’ ranged from 35 to 55% and varied from 0 to 4% per item for the response option ‘strongly disagree’. The percentage of participants that used the response option ‘not applicable’ varied from 1 to 8% per item. There were no missing values at time 2.

Mean scores ranged between 4 and 4.37 with a mean of 4.2 and mean SD = 0.9. Over 90% of participants felt confident managing their care at home and knew who to contact and what to do in an emergency. However, one in ten patients felt they could not manage their overall health and care safely at home, and did not feel they knew how to manage their medication. Similarly, one in ten patients reported they did not feel they could talk to health care professionals or a family member about their worries and did not feel supported by their community health care services to manage both medical and non-medical needs (e.g. cooking, cleaning, buying food, dressing).

**Table 3:** Means, standard deviations and frequencies of PACT-M 2 items

**PACT-M 2** ***n*** **= 110**	**Strongly Disagree (%)**	**Disagree (%)**	**Neither Agree nor Disagree (%)**	**Agree (%)**	**Strongly Agree (%)**	**Not Applicable (%)**
1. I know who to contact if I have any questions around my health and healthcare.	1	3	3	47	46	1
2. I know how to manage my medicines.	**2**	5	2	41	49	2
3. I have the necessary support to manage everyday activities (e.g. cooking, cleaning, buying food, showering, walking, dressing).	4	5	2	36	46	9
4. I feel I have the support I need from community health services, (e.g. doctors, nurses, home care staff).	3	8	6	47	31	6
5. I feel confident about managing my health at home.	1	4	6	45	45	1
6. I feel that there is someone I can talk to about my worries (for example, health care staff or my family).	3	4	4	45	42	4
7. I know what to do and who to contact if my health gets worse.	1	1	2	47	48	1
8. I feel I can now manage my care safely at home	3	5	4	47	41	1

### Problems with healthcare and adverse events

We sought to capture potential problems with care and adverse events by asking participants if they have experienced a list of health related problems. At all follow up points, we asked participants to respond whether they have had any health problem, since their latest discharge from hospital (Table 4).

We asked participants to report any problems they had since their latest admission (rather than since we last spoke to them) to ensure that we will capture the experiences of people that have been readmitted to the hospital in an unplanned manner. The most common problems within the first week post discharge were; emergency appointments with local or hospital services, problems with managing medication and infections. Within the first month post discharge, people most often experienced problems with medication management, having difficulties arranging appointments with health care professionals and visiting health services in an unplanned manner.

**Table 4:** Problems with care and adverse events at time 1 (T1) and 2 (T2)

	**T1 (%)**	**T2 (%)**
1. Have you had any sores that won’t heal?	4	9
2. Have you had any infections?	14	19
3. Have you had a fall?	8	8
4. Have you had any difficulty getting an appointment with a doctor or other healthcare person?	11	19
5. Have you had any problems with your medication?	15	9
6. Have you had any problems getting essential healthcare supplies (like pads or prescribed feed)?	1	3
7. Have you had any additional problems that led to contacting the GP (not routine) or anyone else, or go to the hospital or emergency services?	25	33

#### Problems with healthcare and adverse events at Time 1: within one week post discharge

One in four participants reported having visited the emergency services or their local surgery/community service in an unplanned manner within the first week after they were discharged. One in six participants reported having issues managing their medication and having had an infection since their discharge. Fewer people (11%) had difficulties accessing their local health professionals and even less patients reported having experienced a fall (8%), issues with pressure ulcers (4%) or having difficulty accessing essential healthcare supplies (1%).

Half of participants reported suffering at least one transition related safety event within seven days after their discharge from hospital. One in four patients experienced between 2 and 3 health issues and 2% experienced four health problems.

#### Problems with healthcare and adverse events at Time 2: within one week postt discharge

One in three patients reported having visited the emergency services or their local surgery/community service in an unplanned manner one month after their discharge. One in five reported having acquired an infection and having difficulty arranging appointments with their local healthcare practitioners. One in ten participants experienced medication management problems (including accessing and taking medication) and suffered from sores that would not heal (for example, pressure ulcers). Fewer people experienced health problems such as having had a fall (8%) and having difficulty accessing essential healthcare supplies (3%).

Sixty-percent of this study’s participants reported having experienced at least one transition related safety event with their care after one month of their discharge from hospital to their home. 25% patients experienced between two and three health related issues and 2% experienced 4 or 5 health problems.

Respondents reported having experienced more problems with their care at one month post discharge (compared to one week post discharge) as five out of seven health related problems increased between 30 and 200% at Time 2. The problem that increased the most at time 2 was getting essential healthcare supplies. Issues with falls remained the same across both time points.

### Exploratory scale structure

Given the low amount of ‘not applicable’ responses (between 0 and 33% for time 1 and 1 to 8% for time 2), for all the exploratory and confirmatory factor analyses we performed, we treated ‘not applicable’ responses as missing values so that the ‘not applicable’ answers would not be included in the analysis.

#### PACT-M 1: preparation for transition

We conducted an exploratory factor analysis using the principal components analysis extraction method on the 9 PACT-M 1 items to determine the factor structure. The Kaiser-Meyer-Olkin measure verified the sampling adequacy for the analysis; KMO = 0.8, above the commonly recommended value of .6, and Bartlett’s test of sphericity was significant (x^2^ (138) = 240 *p* < .001). KMO values for individual items were above the acceptable limit of 0.5 [47, 48]. Finally, the communalities were between 0.2 and 0.6 (Table 5), further confirming that each item shared some common variance with other items. These overall indicators showed that correlations between items were sufficiently large for factor analysis [42, 47, 48].

Using Kaiser’s criterion, the analysis revealed two factors with eigenvalues greater than 1 (4.1, 1.1), with the first factor explaining 45% of the variance and the second a further 13%. The scree plot showed inflexions suggesting one factor structure. The component matrix showed strong loadings of all items on the first factor, with values higher than 0.6 (above the recommended value of 0.3) (Table 5). Only one item (item 2) loaded strongly on the second factor (0.7) and showed good loading value on the first factor (0.5). These results implied a unidimensional factor model of the PACT-M 1.

A number of criteria are used to determine a single dimension of the scale; (i) ratio of first to second eigenvalues was equal or larger than 3, (ii) the variance explained by the first factor to be greater than 20%, (iii) eigenvalues accounting for 10% of variance and greater than 1 and (iv) examining the inflection point on the scree Test [49, 50]. Given the first to second eigenvalue ratio (=3), the high variance explained by the first factor (eigen value> 1), the scree plot presentation and the strong factor loadings of the majority of items on the first factor, we sought to explore the unidimensionality of the PACT-M 1 and conducted a confirmatory factor analysis retaining a single factor assumption.

**Table 5:** PACT-M 1: Cronbach’s alpha, communalities and factor loadings

	Cronbach’s Alpha	Eigen Value	Variance %	Communalities	Factor Loadings
	0.84	4.1	45		
1. I felt I could ask staff questions about what will happen after going home.				0.6	0.8
2. Before leaving the hospital, I was confident I understood how to manage my medication.				0.2	0.5
3. While I was in hospital, staff helped me to prepare for things that I might find difficult when I go back home (such as walking, cooking, showering, grocery shopping or being in pain).				0.4	0.7
4. Before leaving the hospital, I understood how to get help (or support) from my community services (e.g. doctors, nurses, home care staff).				0.5	0.7
5. Before leaving the hospital, I knew what arrangements had been made to support me at home (for example home care, community care visits).				0.5	0.7
6. While I was in hospital, there was someone who I could talk to if I was worried.				0.5	0.7
7. Before leaving the hospital, I felt confident about what to do if my health became worse at home.				0.4	0.7
8. I feel that my concerns around my health had been addressed before I went home.				0.5	0.7
9. I feel prepared to be at home.				0.3	0.6

Extraction method: principal components analysis

To explore the internal reliability of the single factor post confirmatory factor analysis, Cronbach alpha was calculated for the PACT-M 1 (where ≥0.70 was considered ‘good’) and average inter-item correlations were explored. Reliability analysis showed an alpha value of 0.84, conveying high internal consistency which can further support the single factor solution (Table 5). PACT-M 1 demonstrated ‘good’ internal reliability with average inter-item correlations ranging between the optimal recommendations; 0.2 to 0.6 with the exception of Item 2 showing lower values than 0.2 when correlated with items 3 and 4 (see Supplementary file 3, Table 3).

#### PACT-M 2: managing health and care at home

Principal component analysis was conducted on the 8 PACT-M 2 items. The Kaiser-Meyer-Olkin measure verified the sampling adequacy for the analysis, KMO = 0.9 and Bartlett’s test of sphericity was significant (x^2^ (110) =509.7, *p* < .001). All KMO values for individual items were > .80 and the communalities were above 0.6 for 7 out of 8 items (see Table 6), indicating high common variance between items. These overall indicators showed that correlations between items were sufficiently large for factor analysis.

Using Kaiser’s criterion, the analysis revealed one factor with eigenvalue greater than 1 (5.1), explaining 64% of the variance. The scree plot clearly showed a single factor structure with strong factor loadings; all values higher than .6 (Table 6). The internal consistency of the one factor solution was excellent (Cronbach’s alpha = 0.92).

**Table 6:** PACT-M 2: Cronbach’s alpha, communalities and factor loadings

	Cronbach’s Alpha	Eigen Value	Variance %	Communalities	Factor Loadings
	0.92	5.1	64		
1. I know who to contact if I have any questions around my health and healthcare.				0.6	0.7
2. I know how to manage my medicines.				0.4	0.6
3. I have the necessary support to manage everyday activities (e.g. cooking, cleaning, buying food, showering, walking, dressing).				0.7	0.9
4. I feel I have the support I need from community health services, (e.g. doctors, nurses, home care staff).				0.7	0.9
5. I feel confident about managing my health at home.				0.7	0.9
6. I feel that there is someone I can talk to about my worries (for example, health care staff or my family).				0.6	0.8
7. I know what to do and who to contact if my health gets worse.				0.6	0.8
8. I feel I can now manage my care safely at home				0.8	0.9

Extraction method: principal components analysis

Reliability analysis showed an alpha value of 0.92, conveying high internal consistency which can further support the single factor solution. PACT-M 2 demonstrated excellent internal reliability with inter-item correlations ranging between the optimal recommendations; 0.4 to 0.8 (see Supplementary file 4, Table 4).

## Discussion

This study explored the reliability and validity of the PACT-M, a tool measuring older patients’ experience during two time points; (i) patients’ experience of preparation for discharge using PACT-M 1 within a week of discharge and (ii) coping at home using PACT-M 2 at one month post discharge. Analysis of the factor structure revealed that both PACT-M 1 and PACT-M 2 showed unidimensional scale characteristics with good levels of internal consistency and reliability. As far as we are aware, the PACT-M is the first to evaluate care transition quality and safety relevant to UK population and can be a suitable marker of the quality of care transitions measuring both pre and post hospital experience.

Although respondents reported feeling prepared for their upcoming discharge for 6 out of 9 discharge arrangements, as many as 20% did not have key information to help continuity of care and half of participants experienced at least one transition related problem with their care within the first week after discharge. Some of participants (12–20%) were not aware of what (if any) arrangements have been made to support them at home, including clinical (e.g. linking patients to community care) or non-clinical needs (e.g. dressing, showering grocery shopping). These findings are consistent with a previous study which sought to measure continuity of care for older patients one month post discharge; the study found that one in five patients they surveyed, felt they had not been provided with information on their diagnosis and treatment requirements, did not have a follow-up plan and felt they have not been involved in discharge preparations [18].

Participants reported feeling confident about managing their medication just before leaving the hospital as well as one month after discharge. However, 1 in 7 experienced problems managing their medication after they returned home both within one week and within a month after discharge. One explanation about this discrepancy might be the complexity of the item phrasing; ‘managing’ medications is an inclusive statement that could involve several aspects including; which medications, when and how to take them and accessing prescriptions. Patients might feel knowledgeable about how to take their medication just before leaving the hospital but management might become more complicated when being at home, as they need to understand, take and access their medication on their own.

The discrepancy between understanding management of medication at hospital but experiencing medication related problems when being at home, might reflect that older patients may feel gratitude to the systems of care and be less likely to raise concerns [51]. Rydeman and Törnquist [52] reported that older people who felt unprepared to manage their care at home were more likely to accept their discharge when they perceived hospital staff as stressed or overburdened.

Participants reported having an overall good and safe transition process one month after discharge. However, our data showed that 60% of surveyed patients experienced at least one problem with their care or adverse event one moth post discharge. Our results also showed that patients are more likely to be readmitted or visit their local health services in an unplanned manner within their first month after discharge rather than within the first week post discharge.

### Validation of the PACT-M

In line with Cook’s recommendations for evaluating a clinical instrument’s validity, we discuss the PACT-M validation process in the context of a broad, established argument that assess validity as reflecting “the degree to which a score can be interpreted as representing the intended underlying construct” [53, 54]. This integrated concept of ‘construct validity’ integrates information from five key areas: content, response process, internal structure, relations to other variables and consequences [54]. The first three areas of validity are addressed in present and previous studies, and the last two will be explored more fully in future work.

#### Content

The content criterion refers to the relationship between the tool’s content and the underlying construct it intends to measure. We used an evidence-based instrument development process to define the theoretical construct that would underline the PACT-M. We achieved that by reviewing existing measures of care transitions and interventions designed to improve transitions for older adults. We then reviewed and incorporated learnings from qualitative studies that explored patients’ and clinical professional’s views and experiences in order to identify key areas of care transition [38]. Onwards, generated an item pool after incorporating feedback from a Patient and Public Involvement (PPI) panel, followed by refining items using a modified Delphi process, to examine content validity of the draft measure [38]. The full PACT-M development process has been described elsewhere [38]. This integrated approach enabled us to identify the full spectrum of transition components, relevant to older patients.

The majority of the PACT-M responses were received over telephone administration. Once contact had been made, participants were almost invariably willing to complete the short interviews, suggesting that both the format and actual questions are understandable and appropriately framed. Telephone administered surveys that include easily understood items, contain middle options on the response scales, are pilot tested with a small sample of the target population and make the necessary adjustments to the wording, can ensure minimal non-responses [39, 55]. Having established these steps during the PACT-M development process [38] we believe that the content of the PACT-M is relevant to the target population and includes items that are easy and quick to respond to.

#### Response process

This process explores and establishes the researchers’ or observers’ level of understanding of the instrument’s theoretical construct. We designed a training manual for the PACT-M administration which spanned from screening patients and recruitment activities, to the PACT-M administration and following up of participants at all time points, with specific instructions on managing attrition and withdrawal incidences. The training instructions were discussed with all researchers and clinicians involved in recruitment activities. Onwards, we organised consultation sessions with 5 members of our PPI panel in order to explore and ratify the researchers’ understanding of the PACT-M overall format, individual items and scoring system. We then administered the measure in person and by telephone. We also collected feedback on the questionnaire administration from the researcher involved in the pilot testing (please see measure development paper [38] for more details on pilot testing) and discussed personal reflections of the measure administration in roundtable discussions with the project team [38].

This integrated process ensured a more robust measure administration technique that improved overall follow up rates during formal testing of the PACT-M; attrition at time 1 during the pilot stage was 46%, dropped to 25% at time 1 and was further reduced to 15% at time 2 during formal testing [38].

#### Internal structure

Evidence on the internal structure of the PACT-M derived from internal consistency analysis, reliability statistics, and factor structure. Reliability and validity of the scales was considered satisfactory; reliability analysis of PACT-M 1 and 2 showed alpha values greater than 0.8 conveying high internal consistency. Both PACT-M versions demonstrated ‘good’ internal reliability with average inter-item correlations meeting the optimal recommendations. This means that the PACT-M produced valid and consistent results across all items. The factor analysis revealed a single factor solution with good factor loadings for all items.

The PACT-M has shown evidence to suggest that it is a reliable measure to capture patients’ perception of the quality of discharge arrangements that can reflect on patients’ ability to manage their care at home further down their care journey. Research findings indicate that patients have a deep understanding of the strengths and deficiencies of health services they are provided with and they are able to identify errors that professionals may fail to notice [56], report safety incidents that may have been overlooked [11] and identify factors related to adverse events [57–59]. Lower item scores in either component of PACT-M may help clinicians identify areas that could be improved.

#### Relationship to other measures

In order to assess the relationship between other measures, PACT-M scores need to be correlated with scores from other similar instruments assessing the same underlying construct. We do not yet have evidence of comparing the PACT-M variables to other scoring systems but that would be an area for future research. This should be done with caution as most measures assessing discharge care only address one area of the transition process at one time point [1, 18, 33] whereas the PACT-M evaluates patient experience across a broader transition time span. Boge et al. [30], recently developed a measure to assess quality of discharge process in older patients called the Discharge Care Patient Experience Survey (DICARES). Validity testing showed a three factor structure distinguishing between items referring to discharge and post discharge care which is in line with our two-time point approach of measure administration. However, in this study, all surveys were completed while patients were in hospital, which suggests a limited insight into the full range of care transition.

#### Consequences

The validity criterion of ‘consequences’ refers to the outcomes of the tool implementation; for example, whether the instrument has contributed to changing health services delivery or improved quality of patient care. We have provided evidence that the PACT-M can be used as a metric for discharge and continuity of care quality and can be used to identify areas for improvement of services targeted to care transitions. The present research is one of five work streams that collectively aim to gain a more insightful understanding of care transitions from patients’ [25] and clinical professionals’ [60] point of view. We will evaluate the PACT-M implementation as part of the intervention evaluation process that will be conducted by our fellow researchers of this programme of work, through a randomised controlled trial, which will provide evidence of the role and impact of PACT-M in the wider context of healthcare improvement.

### Strengths and limitations

The main strength of the study is the development of a valid and reliable tool that can be used to improve safety at care transitions. The PACT-M showed it can be a reliable indicator of quality and safety of care at two time points; at preparation for discharge and coping at home. An additional benefit of the PACT-M is that it is quick and not burdensome for patients to complete. Furthermore, the PACT-M tool is the only available measure that collects patient feedback on the experience of care transition at the immediate and longer post discharge period. This study has some limitations. Study participants were English speaking with cognitive capacity to consent, mostly of Caucasian ethical background. Non-English speaking older people unable to compete the PACT-M were not included in the study. Patients who were unable to speak English, unable to read and write or unable to complete the questionnaire were excluded. Thus, the findings may not be applicable to the potentially more vulnerable populations who may have difficulties communicating. Future studies should investigate ways to use the PACT-M in trials including a more diverse population, especially older more vulnerable people who live alone.

## Conclusion

The results of the study suggest that the PACT-M shows acceptable validity and reliability. Further research is needed to assess the test-retest reliability criterion, as well as the PACT-M’s usability by a patient’s proxy, for example, a caregiver. Future research should explore the PACT-M validity by comparing the PACT-M with other similar scoring systems. Health services providers aiming to evaluate and improve transition quality may use the PACT-M to identify areas for improvement; by exploring the number of patients that disagree with the statements included in the PACT-Mat the point of discharge and at the immediate and longer term post-discharge period.

## Supplementary information

**Additional file 1. **Table 1. PACT-M 1 items.

**Additional file 2. **Table 2. PACT-M 2 items.

**Additional file 3. **Table 3. Inter-item correlations PACT-M 1, *n* = 138.

**Additional file 4. **Table 4. Inter-item correlations PACT-M 2, *n* = 110.

## Data Availability

The datasets used and analysed during the current study are available from the corresponding author on reasonable request.

## Abbreviations

PACT-M: Partners at Care Transitions Measure
NHS: National Health Service
GP: General Practitioner
CTM: Care Transitions Measure
PPI: Patient and Public Involvement
