# Supplementary material for: Validation of the Partners at Care Transitions Measure (PACT-M): assessing the quality and safety of care transitions for older people in the UK
Source: BMC Health Serv Res. 2020 Jul 1;20:608. doi: 10.1186/s12913-020-05369-1 (PMC7329420; doi:10.1186/s12913-020-05369-1)
Supplement: Supplementary file 1 — Additional file 1. Table 1. PACT-M 1 items. [file 12913_2020_5369_MOESM1_ESM.docx]

Supplementary file 1

Table 1. *PACT-M 1 items.*

|  | **Strongly Disagree** | **Disagree** | **Neither Agree nor Disagree** | **Agree** | **Agree Strongly** | **Don't Know/Don't Remember/Not Applicable** |
| --- | --- | --- | --- | --- | --- | --- |
| 1. I felt I could ask staff questions about what will happen after going home. |  |  |  |  |  |  |
| 2.    Before leaving the hospital I was confident I understood how to manage my medication. |  |  |  |  |  |  |
| 3.    While I was in hospital, staff helped me to prepare for things that I might find difficult when I go back home (such as walking, cooking, showering, grocery shopping or being in pain). |  |  |  |  |  |  |
| 4.    Before leaving the hospital, I understood how to get help (or support) from my community services (e.g. doctors, nurses, home care staff). |  |  |  |  |  |  |
| 5.    Before leaving the hospital I knew what arrangements had been made to support me at home (for example home care, community care visits). |  |  |  |  |  |  |
| 6.    While I was in hospital, there was someone who I could talk to if I was worried. |  |  |  |  |  |  |
| 7.    Before leaving the hospital, I felt confident about what to do if my health became worse at home. |  |  |  |  |  |  |
| 8.   I feel that my concerns around my health had been addressed before I went home. |  |  |  |  |  |  |
| 9. I feel prepared to be at home. |  |  |  |  |  |  |
