# Supplementary material for: Validation of the Partners at Care Transitions Measure (PACT-M): assessing the quality and safety of care transitions for older people in the UK
Source: BMC Health Serv Res. 2020 Jul 1;20:608. doi: 10.1186/s12913-020-05369-1 (PMC7329420; doi:10.1186/s12913-020-05369-1)
Supplement: Supplementary file 2 — Additional file 2. Table 2. PACT-M 2 items. [file 12913_2020_5369_MOESM2_ESM.docx]

Supplementary file 2

Table 2. *PACT-M 2 items.*

|  | **Strongly Disagree** | **Disagree** | **Neither Agree nor Disagree** | **Agree** | **Agree Strongly** | **Don't Know/Don't Remember/Not Applicable** |
| --- | --- | --- | --- | --- | --- | --- |
| 1.    I know who to contact if I have any questions around my health and healthcare. |  |  |  |  |  |  |
| 2.    I know how to manage my medicines. |  |  |  |  |  |  |
| 3.    I have the necessary support to manage everyday activities (e.g. cooking, cleaning, buying food, showering, walking, dressing). |  |  |  |  |  |  |
| 4.    I feel I have the support I need from community health services, (e.g. doctors, nurses, home care staff). |  |  |  |  |  |  |
| 5.    I feel confident about managing my health at home. |  |  |  |  |  |  |
| 6.    I feel that there is someone I can talk to about my worries (for example, health care staff or my family). |  |  |  |  |  |  |
| 7.    I know what to do and who to contact if my health gets worse. |  |  |  |  |  |  |
| 8.    I feel I can now manage my care safely at home |  |  |  |  |  |  |
