# Supplementary material for: Validation of the Partners at Care Transitions Measure (PACT-M): assessing the quality and safety of care transitions for older people in the UK
Source: BMC Health Serv Res. 2020 Jul 1;20:608. doi: 10.1186/s12913-020-05369-1 (PMC7329420; doi:10.1186/s12913-020-05369-1)
Supplement: Supplementary file 3 — Additional file 3. Table 3. Inter-item correlations PACT-M 1, n = 138. [file 12913_2020_5369_MOESM3_ESM.docx]

Supplementary file 3

Table 3. *Inter-item correlations PACT-M 1, n=138.*

|  | | PACT-M1-1 | | PACT-M1-2 | | PACT-M1-3 | | PACT-M1-4 | | PACT-M1-5 | | PACT-M1-6 | | PACT-M1-7 | | PACT-M1-8 | | PACT-M1-9 | |
| --- | --- | --- | --- | --- | --- | --- | --- | --- | --- | --- | --- | --- | --- | --- | --- | --- | --- | --- | --- |
| PACT-M 1 - 1. I felt I could ask staff questions about what will happen after going home. | | 1.0 | |  | |  | |  | |  | |  | |  | |  | |  | |
| PACT-M 1 - 2. Before leaving the hospital, I was confident I understood how to manage my medication. | | 0.4 | | 1.0 | |  | |  | |  | |  | |  | |  | |  | |
| PACT-M 1 - 3. While I was in hospital, staff helped me to prepare for things that I might find difficult when I go back home (such as walking, cooking, showering, grocery shopping or being in pain). | | 0.5 | | 0.1 | | 1.0 | |  | |  | |  | |  | |  | |  | |
| PACT-M 1 - 4. Before leaving the hospital, I understood how to get help (or support) from my community services (e.g. doctors, nurses, home care staff). | | 0.4 | | 0.1 | | 0.5 | | 1.0 | |  | |  | |  | |  | |  | |
| PACT-M 1 - 5. Before leaving the hospital, I knew what arrangements had been made to support me at home (for example home care, community care visits). | | 0.5 | | 0.2 | | 0.5 | | 0.6 | | 1.0 | |  | |  | |  | |  | |
| PACT-M 1 - 6. While I was in hospital, there was someone who I could talk to if I was worried. | | 0.6 | | 0.4 | | 0.5 | | 0.3 | | 0.5 | | 1.0 | |  | |  | |  | |
| PACT-M 1 - 7. Before leaving the hospital, I felt confident about what to do if my health became worse at home. | | 0.4 | | 0.4 | | 0.2 | | 0.5 | | 0.4 | | 0.3 | | 1.0 | |  | |  | |
| PACT-M 1 - 8. I feel that my concerns around my health had been addressed before I went home. | | 0.6 | | 0.4 | | 0.5 | | 0.4 | | 0.4 | | 0.4 | | 0.3 | | 1.0 | |  | |
| PACT-M 1 - 9. I feel prepared to be at home. | 0.3 | | 0.2 | | 0.4 | | 0.4 | | 0.4 | | 0.2 | | 0.5 | | 0.2 | | 1.0 | |  |
