# Supplementary material for: Validation of the Partners at Care Transitions Measure (PACT-M): assessing the quality and safety of care transitions for older people in the UK
Source: BMC Health Serv Res. 2020 Jul 1;20:608. doi: 10.1186/s12913-020-05369-1 (PMC7329420; doi:10.1186/s12913-020-05369-1)
Supplement: Supplementary file 4 — Additional file 4. Table 4. Inter-item correlations PACT-M 2, n = 110. [file 12913_2020_5369_MOESM4_ESM.docx]

Supplementary file 4

Table 4. *Inter-item correlations PACT-M 2, n=110.*

|  | PACT-M2-1 | PACT-M2-2 | PACT-M2-3 | PACT-M2-4 | PACT-M2-5 | PACT-M2-6 | PACT-M2-7 | PACT-M2-8 |
| --- | --- | --- | --- | --- | --- | --- | --- | --- |
| PACT-M 2 - 1. I know who to contact if I have any questions around my health and healthcare. | 1.0 |  |  |  |  |  |  |  |
| PACT-M 2 - 2. I know how to manage my medicines. | 0.4 | 1.0 |  |  |  |  |  |  |
| PACT-M 2 - 3. I have the necessary support to manage everyday activities (e.g. cooking, cleaning, buying food, showering, walking, dressing). | 0.5 | 0.4 | 1.0 |  |  |  |  |  |
| PACT-M 2 - 4. I feel I have the support I need from community health services, (e.g. doctors, nurses, home care staff). | 0.6 | 0.5 | 0.7 | 1.0 |  |  |  |  |
| PACT-M 2 - 5. I feel confident about managing my health at home. | 0.6 | 0.5 | 0.7 | 0.6 | 1.0 |  |  |  |
| PACT-M 2 - 6. I feel that there is someone I can talk to about my worries (for example, health care staff or my family). | 0.6 | 0.5 | 0.6 | 0.7 | 0.6 | 1.0 |  |  |
| PACT-M 2 - 7. I know what to do and who to contact if my health gets worse. | 0.6 | 0.5 | 0.6 | 0.6 | 0.6 | 0.6 | 1.0 |  |
| PACT-M 2 - 8. I feel I can now manage my care safely at home. | 0.6 | 0.5 | 0.8 | 0.7 | 0.9 | 0.6 | 0.6 | 1.0 |
